# Supplementary material for: Serum proteomics study on cognitive impairment after cardiac valve replacement surgery: a prospective observational study
Source: PeerJ. 2024 Jun 18;12:e17536. doi: 10.7717/peerj.17536 (PMC11192023; doi:10.7717/peerj.17536)
Supplement: Supplemental Information 1 [file peerj-12-17536-s001.docx]

**Supplementary tables**

**Table S1**. Demographic characteristics of healthy control group and surgical group

were compared before surgery

| **Parameter** | **Healthy control group**  **(n=50)** | **Surgical group**  **(n=226)** | ***P*-value** |
| --- | --- | --- | --- |
| **Gender** (%) |  |  | 0.755 |
| Female/Male | 23/27(46.0) | 111/115(49.1) |  |
| **Age** (y) (mean±SD) | 55.26±6.59 | 56.44±7.74 | 0.317 |
| **BMI** (mean±SD) | 22.00±3.36 | 23.17±4.12 | 0.072 |
| **Education** (%) |  |  | 0.428 |
| 3 years | 9(18.0) | 64(28.3) |  |
| 6 years | 16(32.0) | 59(26.1) |  |
| 9 years | 18(36.0) | 82(36.3) |  |
| 12 years | 6(12.0) | 16(7.1) |  |
| 15 years or more | 1(2.0) | 5(2.2) |  |
| **MMSE (before surgery)** | 27.74±1.97 | 27.40±1.71 | 0.260 |

**Table S2**. MMSE and MoCA Scores at different time points

|  | **MMSE** | | **MoCA** | |
| --- | --- | --- | --- | --- |
|  | group non-P  (n=153) | group P’ (n=31) | group non-P (n=153) | group P’  (n=31) |
| **Before surgery** | 27.43±1.64 | 27.22±1.98 | 25.51±1.72 | 25.42±1.29 |
| **2d after surgery** | 27.05±1.72 | 23.55±1.41^＊#^ | 25.41±1.93 | 22.74±1.77^＊#^ |
| **Before discharge** | 28.25±1.43^#^ | 25.32±1.35^＊#^ | 25.90±1.78 | 21.97±1.70^＊#^ |
| **6w after surgery** | 28.46±1.38^#^ | 26.13±1.20^＊#^ | 26.20±1.27^#^ | 22.00±1.71^＊#^ |

Note: *In comparison to the group non-P, *P* < 0.01.

#Compared to before surgery, *P* < 0.01.

**Table S3**. Characteristics of patients before surgery

| **Parameter** | **group non-P(n=153)** | **group P’ (n=31)** | ***P*-value** |
| --- | --- | --- | --- |
| **Gender (%)** |  |  |  |
| Female/Male | 72/81 | 11/20 | 0.076 |
| **Age (y) (%)** |  |  | 0.043^＊^ |
| 45~54 | 78(51.0) | 10(32.3) |  |
| 55~64 | 49(32.0) | 10(32.3) |  |
| 65~75 | 26(17.0) | 11(35.5) |  |
| **BMI** (mean±SD) | 22.02±3.46 | 22.00±3.10 | 0.974 |
| **Education** (%) |  |  | 0.439 |
| 3 years | 44(28.8) | 9(29.0) |  |
| 6 years | 45(29.4) | 6(19.4) |  |
| 9 years | 55 (35.9) | 12 (38.7) |  |
| 12 years | 6(3.9) | 3 (9.7) |  |
| 15 years or more | 3 (2.0) | 1(3.2) |  |
| Hypertension |  |  |  |
| No/Yes | 136/17 | 26/5 | 0.630 |
| Atrial fibrillation |  |  |  |
| No/Yes | 99/54 | 20/11 | 0.984 |
| Hepatorenal function |  |  |  |
| No/Yes | 138/15 | 28/3 | 1.00 |
| **Smoking** |  |  |  |
| No/Yes (Abstain or quit smoking for more than 3 years) | 20/61 | 2/18 | 0.261 |
| **Drinking** |  |  |  |
| No/Yes (Abstain or quit smoking for more than 3 years) | 53/28 | 13/7 | 0.971 |
| **ASA grade** |  |  |  |
| Ⅱ/Ⅲ grade | 19/134 | 4/27 | 1.00 |
| **MMSE score pre-operation** | 27.43±1.64 | 27.22±1.98 | 0.540 |
| **Preoperative diagnosis** |  |  | 0.196 |
| Congenital heart disease | 3(2.0) | 2(6.5) |  |
| Rheumatic heart disease | 53(34.6) | 5(16.1) |  |
| Valvular inadequacy | 97(63.4) | 24(77.4) |  |
| **Surgical method** |  |  | 0.136 |
| Aortic valve replacement (AVR) | 34(22.2) | 9(29.0) |  |
| Mitral valve replacement (MVR) | 74(48.4) | 12(38.7) |  |
| Tricuspid valve replacement (TVR) | 1(0.7) | 1(3.2) |  |
| AVR+MVR/TVR | 44(28.8) | 8(25.8) |  |
| AVR+MVR+TVR | 0 | 1(3.2) |  |
| **ΔHB** (mean±SD) | 40.67±16.51 | 40.35±18.43 | 0.924 |
| Chronic diseases (at least one of COPD, vascular disease, anemia or arthritis) [31] |  |  |  |
| No/Yes | 125/28 | 19/12 | 0.014^＊^ |
| Preoperative EF value (%)(mean ±SD) | 57.13±10.53 | 56.23±8.38 | 0.663 |

**Table S4**. Characteristics of patients during surgery

| **Parameter** | **group non-P(n=153)** | **group P’**  **(n=31)** | ***P*-value** |
| --- | --- | --- | --- |
| Sufentanil (μg)(mean±SD) | 327.56±55.68 | 348.55±39.52 | 0.047^＊^ |
| Propofol (ml )(mean±SD) | 148.26±35.45 | 153.06±38.55 | 0.499 |
| Midazolam(mg)(mean±SD) | 4.62±1.90 | 5.13±1.38 | 0.160 |
| Etomidate (mg)(mean±SD) | 15.20±3.36 | 16.19±3.28 | 0.132 |
| Dexmedetomidine (%) |  |  |  |
| No/Yes | 98/54 | 15/16 | 0.093 |
| Sevoflurane |  |  |  |
| No/Yes | 91/62 | 17/14 | 0.632 |
| Rocuronium |  |  |  |
| No/Yes | 31/122 | 3/28 | 0.166 |
| Cisatracurium |  |  |  |
| No/Yes | 60/93 | 18/13 | 0.053 |
| Amount of bleeding (ml)  (IQR) | 500.00[400.00, 500.00] | 500.00[500.00, 600.00] | 0.221 |
| Duration of surgery (min)(mean±SD) | 317.34±77.0 | 338.71±92.23 | 0.175 |
| CPB time (min)(mean±SD) | 147.09±46.38 | 165.35±48.11 | 0.048^＊^ |
| Duration of anesthesia  (min)(mean±SD) | 372.10±79.59 | 385.65±93.00 | 0.403 |
| Epinephrine (mg)(IQR) | 0.31[0.21, 0.38] | 0.30[0.18, 0.40] | 0.747 |
| Dopamine (mg) (IQR]) | 31.78[22.00, 40.00] | 27.00[18.00, 44.00] | 0.488 |
| Nitroglycerin(mean±SD) |  |  |  |
| No/Yes | 24/128 | 2/29 | 0.282 |
| Temperature of CPB | 33.01±0.78 | 32.81±0.60 | 0.187 |

Note：*In comparison to the group non-P, *P* < 0.05.

**Table S5**. Characteristics of patients after surgery

| **Parameter** | **group non-P(n=153)** | **group P’**  **(n=31)** | ***P*-value** |
| --- | --- | --- | --- |
| Intubation time (h)  (IQR) | 19.00[15.00, 24.50] | 20.00[16.00, 28.00] | 0.168 |
| Epinephrine (mg)(IQR) | 6.00[4.70, 7.05] | 5.10[3.20, 7.60] | 0.434 |
| Dexmedetomidine (%) |  |  |  |
| No/Yes | 73/80 | 10/21 | 0.115 |
| VAS score | 4.00[4.00, 5.00] | 4.00[4.00, 5.00] | 0.988 |
| Complication **(**pleural effusion**,** cardiac tamponade, arrhythmia. et al.**)** |  |  |  |
| No/Yes | 34/119 | 11/20 | 0.117 |

**Table S6**. MMSE and MoCA scores at different time points after PSM

|  | **MMSE** | | **MoCA** | |
| --- | --- | --- | --- | --- |
|  | group C  (n=25) | group P  (n=25) | group C  (n=25) | group P  (n=25) |
| **Before surgery** | 28.12±1.39 | 27.48±1.90 | 26.44±1.80 | 25.68±1.18 |
| **2d after surgery** | 27.96±1.30 | 23.40±1.44^＊#^ | 25.64±1.58 | 22.36±1.70^＊#^ |
| **Before discharge** | 29.36±0.86^＊^ | 25.56±1.27^＊#^ | 26.76±1.59 | 21.88±1.70^＊#^ |
| **6 w after surgery** | 29.32±0.85^＊^ | 26.08±1.18^＊#^ | 27.12±1.36 | 21.84±1.71^＊#^ |

Note: # Compared to the group C: *P* < 0.01;

* Compared to before surgery, *P* < 0.01.

**Table S7.** Characteristics of patients after PSM

| **Parameter** | **group C(n=25)** | **group P(n=25)** | ***P*-value** |
| --- | --- | --- | --- |
| **Preoperative indictors** |  |  |  |
| **Gender (%)** |  |  |  |
| Female/Male | 10/15 | 8/17 | 0.556 |
| **Age (y) (%)** |  |  | 0.951 |
| 45~54 | 11(44.0) | 10(40.0) |  |
| 55~64 | 9(36.0) | 10(40.0) |  |
| 65~75 | 5(20.0) | 5(20.0) |  |
| **BMI**(mean±SD) | 20.94±2.90 | 22.08±3.40 | 0.209 |
| **Education** (%) |  |  | 0.608 |
| 3 years | 3(12.0) | 6(24.0) |  |
| 6 years | 5(20.0) | 4(16.0) |  |
| 9 years | 14(56.0) | 11(44.0) |  |
| 12 years | 1(4.0) | 3(12.0) |  |
| 15 years or more | 2(8.0) | 1(4.0) |  |
| **ASA grade** |  |  |  |
| Ⅱ/Ⅲ grade | 4/21 | 4/21 | 1.00 |
| **MMSE score pre-operation** | 28.12±1.39 | 27.48±1.90 | 0.181 |
| **Preoperative diagnosis** |  |  | 0.208 |
| Congenital heart disease | 1(4.0) | 2(8.0) |  |
| Rheumatic heart disease | 10(40.0) | 4(16.0) |  |
| Valvular inadequacy | 14(56.0) | 19(76.0) |  |
| **Coronary artery stenosis** |  |  |  |
| No/Yes | 24/1 | 20/5 | 0.189 |
| Preoperative EF value (%) (mean±SD) | 59.29±9.53 | 56.37±8.63 | 0.272 |
| **Hypertension** |  |  |  |
| No/Yes | 24/1 | 22/3 | 0.602 |
| Atrial fibrillation |  |  |  |
| No/Yes | 16/9 | 17/8 | 1.00 |
| **Smoking** |  |  |  |
| No/Yes | 13/12 | 10/15 | 0.395 |
| **Drinking** |  |  |  |
| No/Yes | 14/11 | 15/10 | 0.774 |
| **Surgical method** |  |  | 0.904 |
| AVR | 8(32.0) | 9(36.0) |  |
| MVR | 9(36.0) | 7(28.0) |  |
| TVR | 0 | 1(4.0) |  |
| AVR+MVR/TVR | 8(32.0) | 7(28.0) |  |
| AVR+MVR+TVR | 0 | 1(4.0) |  |
| **Anesthesia drugs** |  |  |  |
| Sufentanil  (μg) (mean±SD) | 340.36±49.53 | 355.00±35.24 | 0.234 |
| Propofol (ml) (mean±SD) | 154.04±27.58 | 156.60±37.25 | 0.784 |
| Etomidate (mg) (mean±SD) | 14.00[12.00,20.00] | 16.00[12.00,20.00] | 0.802 |
| Midazolam (mg) (IQR) | 5.00[5.00, 6.00] | 5.00[4.00, 6.00] | 0.502 |
| Rocuronium |  |  |  |
| No/Yes | 3/22 | 2/33 | 1.00 |
| Cisatracurium |  |  |  |
| No/Yes | 9/16 | 14/11 | 0.156 |
| Epinephrine (mg) (IQR) | 0.27[0.15, 0.37] | 0.30[0.20, 0.45] | 0.241 |
| Dopamine (mg) (mean±SD) | 29.21±13.19 | 32.24±14.42 | 0.478 |
| Nitroglycerin (mg) (mean±SD) | 2.56±1.34 | 2.91±1.29 | 0.378 |
| Sevoflurane (%) |  |  |  |
| No/Yes | 17/8 | 14/11 | 0.561 |
| Dexmedetomidine (%) |  |  |  |
| No/Yes | 15/10 | 13/12 | 0.776 |
| **Intraoperative indicators** |  |  |  |
| Amount of bleeding (ml) (IQR) | 500.00[300.00, 550.00] | 500.00[500.00, 500.00] | 0.600 |
| MAP (mmHg) (mean±SD) | 75.93±4.47 | 74.56±5.29 | 0.328 |
| HR (bmp) (mean±SD) | 94.41±12.20 | 93.59±9.62 | 0.793 |
| BIS (mean±SD) | 47.13±3.48 | 47.57±4.02 | 0.682 |
| CPB time (min) (mean±SD) | 148.28±40.34 | 160.04±51.68 | 0.374 |
| Duration of anesthesia (min)  (mean±SD) | 387.80±88.46 | 399.20±89.29 | 0.625 |
| Duration of surgery (min)  (mean±SD) | 323.60±87.17 | 340.20±90.33 | 0.512 |
| **ΔHB** (mean±SD) | 44.24±14.99 | 40.80±18.86 | 0.479 |
| Temperature of CPB (mean ±SD) | 33.00±0.67 | 33.04±0.59 | 0.833 |
| **Postoperative indicators** |  |  |  |
| Intubation time in ICU (h)  (IQR) | 19.00[14.00, 25.50] | 20.00[16.00, 29.00] | 0.140 |
| Dexmedetomidine (IQR) | 0.60[0.40, 0.60] | 0.67[0.40, 0.80] | 0.310 |
| Dopamine (mg) (IQR) | 540.00[300.00, 900.00] | 600.00[390.00,720.00] | 0.876 |
| Epinephrine (mg) (IQR) | 6.00[2.50, 7.75] | 5.00[2.85, 6.95] | 0.676 |
| VAS score (IQR) | 4.00[4.00, 5.00] | 4.00[4.00, 5.00] | 0.426 |
| Complication **(**pleural effusion**,** cardiac tamponade, arrhythmia. et al.**)** |  |  |  |
| No/Yes | 7/18 | 9/16 | 0.544 |
