# Supplementary material for: Serum proteomics study on cognitive impairment after cardiac valve replacement surgery: a prospective observational study
Source: PeerJ. 2024 Jun 18;12:e17536. doi: 10.7717/peerj.17536 (PMC11192023; doi:10.7717/peerj.17536)
Supplement: Supplemental Information 4 [file peerj-12-17536-s004.docx]

STROBE Statement—checklist of items that should be included in reports of observational studies

|  | Item No. | Recommendation | Page  No. | Relevant text from manuscript |
| --- | --- | --- | --- | --- |
| **Title and abstract** | 1 | (*a*) Indicate the study’s design with a commonly used term in the title or the abstract | Page 2 | A prospective observational study |
|  |  | (*b*) Provide in the abstract an informative and balanced summary of what was done and what was found | Page 2 | The occurrence of PND can impact some oxidative stress proteins. This study provide data for future studies about PND to general anaesthesia and surgeries. |
| Introduction | | | |  |
| Background/rationale | 2 | Explain the scientific background and rationale for the investigation being reported | Page 2 | PND is high, especially after cardiac surgeries, and the underlying mechanisms remain elusive. |
| Objectives | 3 | State specific objectives, including any prespecified hypotheses | Page 2 | we conducted a prospective observational study to observe serum proteomics differences in PND patients after cardiac valve replacement surgery. |
| Methods | | | |  |
| Study design | 4 | Present key elements of study design early in the paper | Page 2 | A prospective observational study |
| Setting | 5 | Describe the setting, locations, and relevant dates, including periods of recruitment, exposure, follow-up, and data collection | Page 3 | Patients who underwent heart valve replacement at Zunyi Medical University Hospital from October 2022 to April 2023 under CPB were included (The first patient in this study was included on October 11th, 2022, and the last visit was on November 24th, 2022) |
| Participants | 6 | (*a*) *Cohort study*—Give the eligibility criteria, and the sources and methods of selection of participants. Describe methods of follow-up  *Case-control study*—Give the eligibility criteria, and the sources and methods of case ascertainment and control selection. Give the rationale for the choice of cases and controls  *Cross-sectional study*—Give the eligibility criteria, and the sources and methods of selection of participants | Page 3 | The inclusion criteria were as follows: Patients between the ages of 45 and 74 who will undergo heart valve replacement surgery under CPB and are classified as ASA grade I- III. The exclusion criteria: cerebrovascular disease, epilepsy, other central nervous system diseases, metabolic disease, history of endocrine disorders, patients with neurological or psychiatric illnesses, preoperative MMSE score ≤ 23, and emergency patients. |
|  |  | (*b*) *Cohort study*—For matched studies, give matching criteria and number of exposed and unexposed  *Case-control study*—For matched studies, give matching criteria and the number of controls per case | Page 4 | Based on Z-scores, patients were categorized into PND group (group P') and non-PND group (group non-P). Using PSM (with four risk factors as covariates and a caliper width of 0.2) to match 1:1 from the group non-PND to create a matched group C that has similar baseline characteristics to the group P' (17). The resulting matched groups are the group P and the group C. |
| Variables | 7 | Clearly define all outcomes, exposures, predictors, potential confounders, and effect modifiers. Give diagnostic criteria, if applicable | Page 5 | age, the presence of chronic disease, the dose of sufentanil, and the duration of CPB are factors influencing the occurrence of PND |
| Data sources/ measurement | 8* | For each variable of interest, give sources of data and details of methods of assessment (measurement). Describe comparability of assessment methods if there is more than one group | *Page4-5* | Two independent samples were compared using Student's t-test or the Mann-Whitney U-test (when variances were homogeneous and non-normal), and chi-square tests or Fisher's exact probability tests were applied for count data. *P* < 0.05 was considered statistically significant. |
| Bias | 9 | Describe any efforts to address potential sources of bias |  |  |
| Study size | 10 | Explain how the study size was arrived at | Page 4 | The postoperative patients were divided into two groups: PND (group P’) and non-PND (group non-P). The incidence rate of PND was evaluated as approximately 24% based on preliminary experiment. The reported incidence of neurocognitive disorders after cardiac valve surgery in Chinese individuals was approximately 33% according to relevant literature. Using the PASS 2021 software (PASS 2021 Power Analysis and Sample Size Software (2021). NCSS, LLC. Kaysville, Utah, USA, ncss.com/software/pass.) and setting the parameters for one proportion, two sides to P0 0.33, P1 0.24, with a significance level of 0.05 and a test power of 0.80, we estimated a drop-out rate of 10% and included 226 subjects in the study. |

Continued on next page

| Quantitative variables | 11 | Explain how quantitative variables were handled in the analyses. If applicable, describe which groupings were chosen and why | Page 4 | Based on Z-scores, patients were categorized into PND group (group P') and non-PND group (group non-P). |
| --- | --- | --- | --- | --- |
| Statistical methods | 12 | (*a*) Describe all statistical methods, including those used to control for confounding | Page 4-5 | Using PSM (with four risk factors as covariates and a caliper width of 0.2) to match 1:1 from the group non-PND to create a matched group C that has similar baseline characteristics to the group P. |
|  |  | (*b*) Describe any methods used to examine subgroups and interactions |  |  |
|  |  | (*c*) Explain how missing data were addressed | Page 5 | A total of 226 patients were enrolled. Two patients were excluded due to perioperative deaths. By the end of 6 weeks, 22 patients had recovered their cognitive ability compared with the second day after surgery (out of 53 patients with PND on the second day post-surgery, 44 on the day before discharge, and 31 by the sixth week). Eighteen cases were lost to follow-up. |
|  |  | (*d*) *Cohort study*—If applicable, explain how loss to follow-up was addressed  *Case-control study*—If applicable, explain how matching of cases and controls was addressed  *Cross-sectional study*—If applicable, describe analytical methods taking account of sampling strategy |  | Using PSM |
|  |  | (*e*) Describe any sensitivity analyses |  |  |
| Results | | | | |
| Participants | 13* | (a) Report numbers of individuals at each stage of study—eg numbers potentially eligible, examined for eligibility, confirmed eligible, included in the study, completing follow-up, and analysed | Page 5 | A total of 226 patients were enrolled. Two patients were excluded due to perioperative deaths. By the end of 6 weeks, 22 patients had recovered their cognitive ability compared with the second day after surgery (out of 53 patients with PND on the second day post-surgery, 44 on the day before discharge, and 31 by the sixth week). Eighteen cases were lost to follow-up. |
|  |  | (b) Give reasons for non-participation at each stage | Page 5 |  |
|  |  | (c) Consider use of a flow diagram | Figure 1 |  |
| Descriptive data | 14* | (a) Give characteristics of study participants (eg demographic, clinical, social) and information on exposures and potential confounders | Table 1 |  |
|  |  | (b) Indicate number of participants with missing data for each variable of interest |  |  |
|  |  | (c) *Cohort study*—Summarise follow-up time (eg, average and total amount) |  |  |
| Outcome data | 15* | *Cohort study*—Report numbers of outcome events or summary measures over time | *Page 5* | Through a univariate analysis of preoperative, intraoperative, and postoperative related indicators for both the non-P and P' groups, it was found that age, the presence of chronic disease, the dose of sufentanil, and the duration of CPB are factors influencing the occurrence of PND |
|  |  | *Case-control study—*Report numbers in each exposure category, or summary measures of exposure |  |  |
|  |  | *Cross-sectional study—*Report numbers of outcome events or summary measures |  |  |
| Main results | 16 | (*a*) Give unadjusted estimates and, if applicable, confounder-adjusted estimates and their precision (eg, 95% confidence interval). Make clear which confounders were adjusted for and why they were included | Tables |  |
|  |  | (*b*) Report category boundaries when continuous variables were categorized |  |  |
|  |  | (*c*) If relevant, consider translating estimates of relative risk into absolute risk for a meaningful time period |  |  |

Continued on next page

| Other analyses | 17 | Report other analyses done—eg analyses of subgroups and interactions, and sensitivity analyses |  |  |
| --- | --- | --- | --- | --- |
| Discussion | | | | |
| Key results | 18 | Summarise key results with reference to study objectives | Page 6 | **4.1 Incidence of PND**  **4.2** **Risk factors for PND**  **4.3** **GSTO1**  **4.4** **IDH1**  **4.5 CAT**  **4.6** **PFN1** |
| Limitations | 19 | Discuss limitations of the study, taking into account sources of potential bias or imprecision. Discuss both direction and magnitude of any potential bias | Page 9 | **Limitations** |
| Interpretation | 20 | Give a cautious overall interpretation of results considering objectives, limitations, multiplicity of analyses, results from similar studies, and other relevant evidence | Page 6-8 | Discussion |
| Generalisability | 21 | Discuss the generalisability (external validity) of the study results | Page 8 | Discussion |
| Other information | |  | | |
| Funding | 22 | Give the source of funding and the role of the funders for the present study and, if applicable, for the original study on which the present article is based | Page 10 | **Zunyi Science and Technology Plan Project (**Grant No: Zunyi City Kehe HZ (2022) 331**) and the Guizhou Provincial Health Commission (**Grant No: **gzwkj2022-129).** |

*Give information separately for cases and controls in case-control studies and, if applicable, for exposed and unexposed groups in cohort and cross-sectional studies.

**Note:** An Explanation and Elaboration article discusses each checklist item and gives methodological background and published examples of transparent reporting. The STROBE checklist is best used in conjunction with this article (freely available on the Web sites of PLoS Medicine at http://www.plosmedicine.org/, Annals of Internal Medicine at http://www.annals.org/, and Epidemiology at http://www.epidem.com/). Information on the STROBE Initiative is available at www.strobe-statement.org.
