# Supplementary material for: Serum proteomics study on cognitive impairment after cardiac valve replacement surgery: a prospective observational study
Source: PeerJ. 2024 Jun 18;12:e17536. doi: 10.7717/peerj.17536 (PMC11192023; doi:10.7717/peerj.17536)
Supplement: Supplemental Information 6 [file peerj-12-17536-s006.docx]

**Rebuttal Letter**

Dear Editor,

I am writing in response to the concern raised regarding the addition of Dr. Yiyong Wei as a co-author in our submitted manuscript. I would like to provide a detailed explanation and justification for this important decision.

Dr. Wei played a pivotal role in this research project, despite his initial reluctance to be included as a co-author due to his extensive publication record. His contributions began during the case collection phase, where he provided invaluable guidance on the psychiatric scale scoring, ensuring the accuracy and reliability of our data. His expertise in this area was crucial in shaping the foundation of our study.

Furthermore, Dr. Wei's involvement extended well beyond the initial data collection. He actively participated in the analysis and selection of differential proteins, a complex and critical step in our research. His insights and expertise were instrumental in identifying key proteins that formed the basis of our findings.

Additionally, Dr. Wei's contributions to the manuscript revision process were significant. He provided thoughtful feedback and suggestions that greatly improved the clarity and impact of our paper. His meticulous attention to detail and commitment to excellence ensured that our research was presented in the best possible light.

Lastly, Dr. Wei's guidance during the submission process was invaluable. His experience and knowledge of the publication landscape were essential in navigating the complexities of submitting our manuscript. His advice and support were critical in ensuring that our research reached the broader scientific community.

Given Dr. Wei's substantial contributions to this project, it is only fitting that he be recognized as a co-author. His expertise, guidance, and support were integral to the success of our research, and his inclusion as a co-author is a small token of our gratitude for his tireless efforts.

We understand the importance of authorship and the need for transparency in research. Therefore, we have carefully considered and documented Dr. Wei's contributions, which clearly meet the criteria for co-authorship. We believe that his inclusion is not only justified but also essential in accurately representing the collaborative nature of our work.

Thank you for considering our explanation. We look forward to a favorable decision on this matter.

Sincerely,

Huanhuan Ma

APR. 1, 2024
